# Sulindac, a non-steroidal anti-inflammatory drug, mediates breast cancer inhibition as an immune modulator

**DOI:** 10.1038/srep19534

**Published:** 2016-01-18

**Authors:** Tao Yin, Guoping Wang, Tinghong Ye, Yongsheng Wang

**Affiliations:** 1 State Key Laboratory of Biotherapy and Cancer Center, West China Hospital, Sichuan University, and Collaborative Innovation Center of Biotherapy, Chengdu 610041, PR China

## Abstract

The cooperation of adaptive immunity with pharmacologic therapy influences cancer progression. Though non-steroidal anti-inflammatory drugs (NSAIDs) have a long history of cancer prevention, it is unclear whether adaptive immune system affects the action of those drugs. In present study, we revealed a novel immunological mechanism of sulindac. Our data showed that sulindac had substantial efficacy as a single agent against 4T1 murine breast cancer and prolonged the survival of tumor-bearing mice. However, in the athymic nude mice, sulindac treatment was ineffective. Further *in vivo* T cell subsets depletion experiments showed that CD8+ T lymphocytes deficiency reversed the anti-tumor effect of sulindac. In addition, sulindac significantly reduced M2 macrophages recruitment, cancer-related inflammation and tumor angiogenesis. Our results advance our understanding of the mechanisms of NSAIDs, and more importantly, this will provide insight into rational drug design or antitumor immunotherapy.

Cyclooxygenases (COXs) pathway is one of the two major enzymatic pathways in arachidonic acid metabolism, the major polyunsaturated fatty acid present in mammalian systems^1^. Prostaglandins and thromboxanes are generated by COXs, which play key roles in inflammation^2^. Besides their anti-inflammatory properties, COXs inhibitors have been used for cancer prevention^3^ and treatment^4^. Sulindac, a non-steroidal anti-inflammatory drug (NSAID) similar to indomethacin, inhibits both COX1 and COX2^5^. Sulindac is metabolized to a pharmacologically active sulfide derivative which inhibits prostaglandin synthesis, and sulfone derivative which lacks prostaglandin synthesis inhibitory activity^6^. Sulindac sulfide is concentrated in the colonic epithelium at concentrations that are at least 20-fold higher than those seen in the serum that are about 10–15 mM^7^, thus sulindac has shown particular efficacy in colon cancers prevention, which could suppress adenoma size and number by as much as 60–70% in patients with familial adenomatous polyposis^8^.

NSAIDs have been investigated in the breast cancer prevention and treatment for decades. Studies of human breast tumor-associated prostaglandins show that high levels of prostaglandin E2 (PGE2) and PGF are associated with the prognosis of patients^9^. Breast cancer risk declined with increasing NSAID exposure^10^. Moreover, high prostaglandin levels are observed to be associated with clinically aggressive behaviors of human breast cancers^11^,^12^. Mouse mammary tumors are heterogeneous in their levels of PGE2 and PGF2a *in situ*, and high PGE levels in these tumors are correlated positively with metastatic potential^13^. Pilot studies in the past couple of years have had promising results in the application of NSAIDs in breast cancer treatment. Oral indomethacin could reduce the growth of transplantable mammary tumors in lymph nodes of Wistar-Furth rats^14^. Continuous administration of indomethacin inhibits both poorly and highly metastatic breast tumors^4^. Inactive sulfone metabolite of sulindac has been reported to inhibit mammary carcinogenesis, especially those with a mutant Ha-ras genotype^15^. However, whether sulindac has a therapeutic efficacy on preexisting breast cancer is unclear.

The well-documented effects of COXs inhibitor on cancer were the inhibition of tumor cells proliferation and induction of apoptosis^16^. However, several lines of evidence suggest that sulindac sulfide could also inhibit the proliferation of cancer cell lines which have no expression of COXs^17^. Furthermore, inactive sulphone derivative of sulindac inhibited tumor cells growth, suggesting the prostaglandin reduction is not necessary for the antineoplastic activity of those drugs^18^. The level of prostaglandin endoperoxide synthase 2 did not predict benefit from aspirin, whereas aspirin use was associated with improved survival in colon cancer patients if tumors expressed HLA class I antigen^19^,^20^. Those reports suggest of alternative mechanisms of COXs inhibitors on tumor inhibition.

Previously, it has been reported that B lymphocytes secrete interleukin-6 (IL-6), IL-4, IL-10 and CXCL1^21^,^22^, which can remold the inflammation in tissues^23^. The activation of B cells results in immunoglobin deposition, angiogenesis, and is essential for epithelial carcinogenesis^24^. As well, T lymphocytes in the tumor microenvironment shape the phenotype of macrophages, which promote breast cancer cells metastasis by producing epithelial growth factors^25^. These results solidify the association of adoptive immune system and tumor progression. Whether adoptive immune system plays roles in the anti-tumor activity of COXs inhibitors has not been explored. Those clues led us to formulate and test the hypothesis that host immune system actively took part in the anti-cancer activity of NSAIDs. Here we found that adaptive immunity intimately affected the pharmacologic action of sulindac on 4T1 murine breast cancer.

## Results

### Sulindac inhibits cancer growth and prolongs survival in immune-competent mice

**Figure 1:**
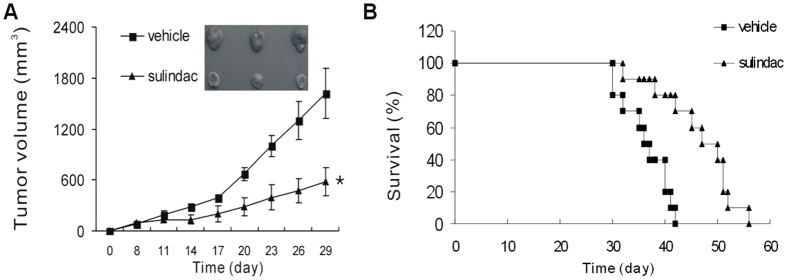
Sulindac inhibited cancer growth and and prolong survival. (**A**) 4T1-bearing mice were treated intraperitoneally with 60 mg/kg sulindac for fourteen consecutive days. Tumor volume was tracked and calculated every three days. *P < 0.05. (**B**) Kaplan-Meyer analysis of mouse survival with a log-rank test. Sulindac leaded to a significant survival advantage. P = 0.001 between two groups.

To investigate whether COXs inhibitor sulindac has a direct anti-tumor effect, 4T1-bearing mice were treated with 60 mg/kg sulindac for consecutive fourteen days. Administration of sulindac triggered rapid 4T1 tumor regression. However, mice that received vehicle did not show any regression (Fig. 1A). Furthermore, mice treated with sulindac had a prolonged survival when compared with vehicle. Mice in sulindac treatment group had a mean survival time of 46.4 ± 2.3 days, while only 36.3 ± 1.4 days in vehicle group (Fig. 1B), suggesting a direct therapeutic efficacy of sulidac as a single agent against 4T1 murine breast cancer.

### Adaptive immunity mediates the anti-tumor effect of sulindac

**Figure 2:**
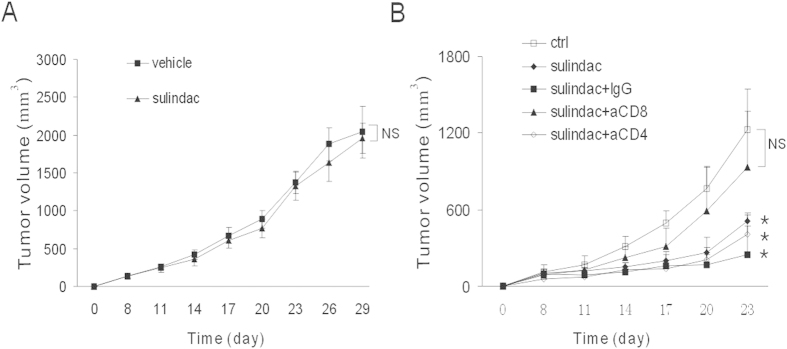
Adaptive immune system mediated the anti-tumor effect of sulindac. (**A**) 4T1 tumor-bearing athymic nude mice were treated intraperitoneally with 60 mg/kg sulindac for fourteen consecutive days. Tumor volume was tracked and calculated every three days. Sulindac was ineffective in immune defective mice. No difference of tumor volume was observed between vehicle and sulindac groups. (**B**) 4T1-bearing BALB/c mice were treated with sulindac, and depleted of CD4+ and CD8+ T cells with GK1.5 and 2.43 antibodies, respectively. IgG group received isotype antibodies. CD8+ T cells depletion reversed the anti-tumor action of sulindac. *P < 0.05 versus control group.

To explore the immunological anti-tumor effect, 4T1 tumors were inoculated into the flank of athymic BALB/c nude mice, which were defective of adoptive immunity. The similar dose of sulidac was given intraperitoneally. Interestingly, almost no effect was observed on athymic mice (Fig. 2A). It was reasonable therefore to conclude that adoptive immunity mediated the anti-tumor effect of sulindac. To more specifically characterize effect of immunity, CD4+ T lymphocytes and CD8+T lymphocytes in 4T1-bearing mice were depleted with GK1.5 and 2.43 antibodies, respectively, followed by sulidac treatment. Fig. 2 showed that CD8 deficiency almost completely blocked the tumor growth retardation of sulindac (Fig. 2B), further validating the inferred function of adaptive immunity on tumor reduction. The anti-tumor effect was not abrogated by depletion of CD4+ T cells. There was also observable tumor inhibition behavior of sulindac by treating with isotype immunoglobin simultaneously (Fig. 2B). Taken together, our findings reveal a pivotal role of CD8+ T lymphocytes in regulating the anti-tumor activity of sulindac.

### Sulindac reduces macrophages influx in tumor microenvironment

**Figure 3:**
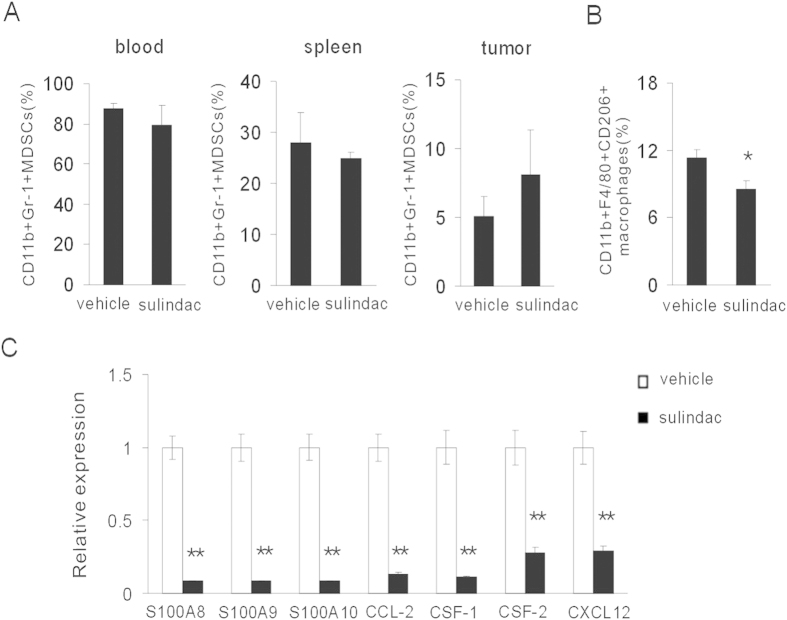
Sulindac reduced macrophages recruitment in tumor microenvironment. (**A**) Seven days after sulindac treatment, CD11b+Gr-1+ MDSCs were evaluated in peripheral blood, spleen and tumors by flow cytometry. (**B**) Quantification of CD11b + F4/80 + CD206 + M2 macrophages was assessed by flow cytometry. There was a reduced number of M2 macrophages after sulindac treatment. *P < 0.05 versus control group. (**C**) The levels of S100A8, S100A9, S100A10, CCL-2, CSF-1, CSF-2 and CXCL12 were analyzed by RT-PCR. Macrophage-associated chemokines were reduced by sulindac. **P < 0.01 versus control group.

The function of CD8+T cells was frequently suppressed in the hostile tumor microenvironment. Based on our intention to mechanistically dissect the role of immunosuppressive cells in tumor regression, we first assessed the quantity of CD11b+Gr1+ MDSCs, a population of well-known immune suppressors, in the blood, spleen and tumor tissues. Flow cytometry results showed that sulidac did not significantly reduce MDSCs in blood, spleen, as well as tumor tissues (Fig. 3A). However, we found that sulindac decreased the number of CD11b+F4/80+CD206+ M2 macrophages (Fig. 3B), indicating an alleviated immune suppressive microenvironment. We further assessed the chemokines expression, which were associated with macrophages recruitment, in the tumor microenvironment. RT-PCR assays revealed that sulindac substantially decreased the expression of S100A8, S100A9, S100A10, CCL-2, CSF-1, CSF-2 and SDF-1/CXCL12, well-known macrophages-associated chemokines (Fig. 3C). We also evaluated the quantity of tumor-infiltrating CD8+ T lymphocytes in the tumor microenvironment. Unexpectedly, tumor regression induced by sulindac was not accompanied by robust infiltration of CD8+ T cells (Supplementary Figure S1A). An unexpected reduction in T cell recruiting chemokines was however measured (Supplementary Figure S1B). Those data suggested that sulindac reduced macrophages influx into tumor microenvironment.

### Sulindac reduces inflammatory and immunosuppressive factors in tumor microenvironment

**Figure 4:**
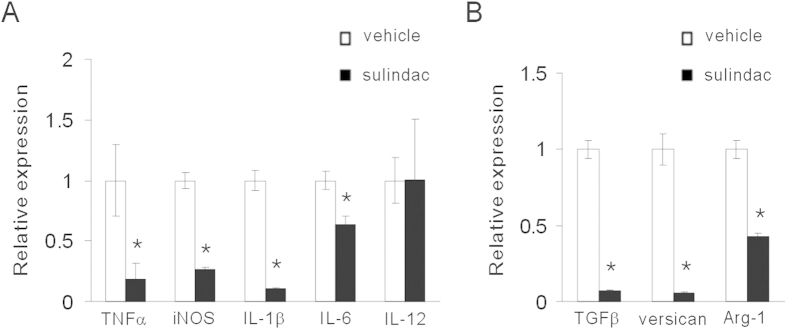
Sulindac reduced pro-inflammatory and immunosuppressive factors in tumor microenvironment. (**A**) The levels of TNFα, iNOS, IL-1β, IL-6 and IL-12 were analyzed by RT-PCR. Sulindac reduced pro-inflammatory factors. *P < 0.05. (**B**) The levels of TGFβ, arginase-1 and versican were evaluated with RT-PCR. Immunosuppressive factors TGFβ, arginase-1 and versican were decreased by sulindac. *P < 0.05.

We next examined the inflammatory factors in the tumor tissues. As expected, tumors exposed to sulindac showed evidence of a decreased inflammatory response. The expression of pro-inflammatory factors TNFα, iNOS, IL-1β and IL-6 were reduced, while IL-12 expression level was similar between two groups (Fig. 4A). Furthermore, we posited that immunosuppressive factors could be reduced by sulindac. Notably, sulindac inhibited TGFβ and arginase-1 expression (Fig. 4B). Versican, an extracellular matrix (ECM) proteoglycan produced by tumor cells, stromal cells and leukocytes, is markedly increased in inflammation and known to stimulate inflammatory cytokine released by immune and inflammatory cells^26^. Of special interest, sulindac remarkably reduced versican expression (Fig. 4B). Those data suggested that sulindac reduced inflammatory and immunosuppressive factors in tumor microenvironment.

### Sulindac reduces tumor vasculature

**Figure 5:**
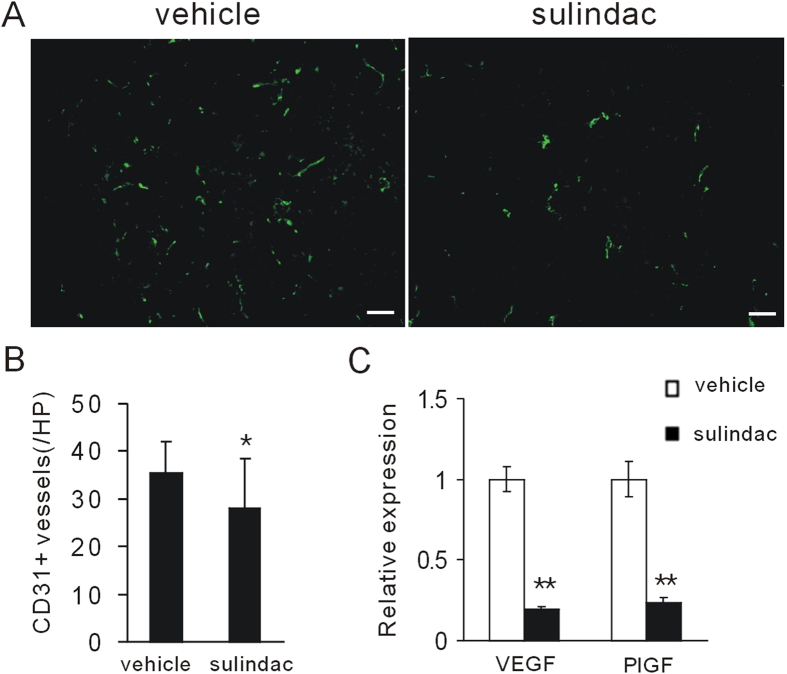
Sulindac decreased tumor vasculature in tumor microenvironment. (**A**) CD31 staining revealed that sulindac reduced tumor angiogenesis in 4T1 tumor model. Scale bars, 50 μm. (**B**) Quantification of tumor vessel densities was assessed. *P < 0.05. (**C**) The level of VEGF and PlGF was evaluated by RT-PCR. Sulindac reduced pro-angiogenic factors VEGF and PlGF. **P < 0.01.

The above data, together with the notion that inflammation and macrophages are associated with tumor angiogenesis^27^,^28^, led us to reason that sulindac might reduce tumor neovascularization. After treatment for seven days, the resulting tumors were excised and examined for histopathology. Microscopic evaluation revealed that sulindac effectively reduced microvessels density in tumor microenvironment (Fig. 5A,B). In addition, sulindac reduced pro-angiogenic factors VEGF and PlGF (Fig. 5C). Those data suggest sulindac could inhibit tumor angiogenesis.

## Discussion

Here we show that the adaptive immune system takes a role in the anti-tumor effect of sulindac. We assessed the therapeutic efficacy of sulindac on 4T1 breast tumor model, which is a commonly used murine breast cancer model, and well-known for its immunosuppressive ability^29^,^30^. Importantly, 4T1 breast cancer cell lines produce PGE2^31^. Up to date, the therapeutic investigation of sulindac has not been described or characterized in 4T1 tumor-bearing mice. Our work adds to and extends prior publications describing the role of NSAIDs in tumor biology. We found that sulindac (60 mg/kg/day) had a potent tumor suppressive effect, which inhibited 4T1 tumor growth by 64% 21 days after treatment beginning. Other studies showed that oral treatment with the same dose of sulindac inhibited V2 carcinoma growth by 35% 17 days after the start of treatment^32^. In Min/+ mice with established intestinal tumors, treatment with sulindac for 4 days reduced tumor number by 75%^33^. In a breast tumor model, continuous oral administration of indomethacin, beginning on the day of breast tumor transplantation, resulted in complete regression of the poorly metastatic, low-PGE, highly immunogenic Tumor 410 in 92% BALB/c mice. Though the high-PGE, highly metastatic, poorly immunogenic Tumor 4501 was partially inhibited by indomethacin, it also increased survival time for tumor-bearing mice (89 versus 53 days for controls)^4^.

The past few decades have seen a groundswell of research on the active involvement of immune system in the therapeutic responses. Many research groups used immune defective mice to evaluate the effects of anti-tumor drugs^34^. EL4 lymphoma were inoculated into Nu/Nu genotype and wide type, respectively, and then treated tumor-bearing mice with chemotherapeutical drugs 5-fluorouracil plus cyclophosphamide. There was observable anti-tumor effect in wide type mice, while no therapeutical effect in nude mice^35^. Antitumor effect of low-dose arsenic trioxide was observed in CT26 tumor-bearing immunocompetent BALB/c mice, and not in nude mice^36^. Gemcitabine, oxaliplatin and mitomycin C have all been shown to exert tumoricidal effects in wide type while not nude mice^37^,^38^,^39^. Severe combined immunodeficient (SCID) phenotype and Rag1−/− mice, lacking functional T and B cells, are also commonly used to assess the immune-drug interactions. In a murine model of acute promyelocytic leukemia (APL), the survival of liposomally encapsulated all-trans-retinoic acid-treated SCID animals is not as long as that of immunocompetent B6C3HF1 recipients^40^. Similarly, small molecule inhibitor imatinib was demonstrated ineffective in Rag−/− mice for gastrointestinal stromal tumor^41^. These insights have formed our current view of immune system in the pharmacology of NSAIDs. As expected, COXs inhibitor sulindac was ineffective in 4T1 tumor-bearing nude mice. Our data provide the first *in vivo* evidence that sulindac is effective for 4T1 murine breast cancer dependent of adaptive immune system. This warrants a better understanding of the molecular mechanisms that underlie NSAIDs.

Several reports demonstrated that the effect of sulindac might not be associated with its COXs inhibition^15^,^42^. We further confirmed the role of CD8+ T lymphocytes. CD4+ T lymphocytes depletion did not affect the action of sulindac. This is a novel new founding of action mechanism of sulindac, in that most of prior works are focused on anti-proliferative activity, apoptosis induction, anti-angiogenesis, and weakening invasiveness effects of those drugs. In addition, more and more evidences show that CD8+ T cells are critical for the anti-tumor activity of many chemical drugs. CD8+ T cells depletion impaired the efficacy of paclitaxel^43^, anthracyclines^44^, imatinib^45^, dasatinib^46^ and BRAF inhibitor PLX4720^47^. Interestingly, in the present study, the anti-tumor effect began to appear less than one week when tumor-bearing mice received sulindac therapy. Further molecular mechanisms should be investigated.

Though CD8+ T cells had a critical role in the anti-tumor effect, in the microenvironment of breast tumors succumbing to sulindac, we did not observed the increased frequency of tumor-infiltrating CD8+ T cells. Thus, our data suggested that the quantity of CD8+ T cells was not entirely responsible for tumor inhibition but, likely, that a complex interdependent network of cells was involved. Our results strongly implicated that the attenuation of immunosuppression might be essential. Notably, sulindac effectively reduced M2 macrophages influx and cancer-related inflammation, which might relieve immune suppression on cytotoxic lymphocytes at some extent. Flow cytometric assessment revealed no differences in MDSCs populations, although this did not rule out any accessory role for these cells in eventual tumor clearance. In addition, the decreased M2 macrophages in the tumor microenvironment contributed to the decreased tumor vasculature by sulindac. Consistent with those results, the levels of VEGF and PlGF were moderately decreased. Our founding is consistent with a recent report that COXs ablation in tumors enables immune attack, and COXs inhibition by aspirin or celecoxib synergizes with checkpoint blockade therapy^31^. As a potent immune modulator, sulindac might be used to design novel tumor immunotherapy strategies.

In summary, our findings underscore the obligate contribution of the adaptive immune system to sulindac-induced breast tumor inhibition. We reveal a novel immunological mechanism of sulindac. More importantly, this will provide insight into rational drug design or antitumor immunotherapy.

## Materials and Methods

### Cell lines and reagents

Murine 4T1 breast cancer cells were obtained from ATCC and were maintained in RPMI-1640 supplemented with 10% fetal bovine serum in a humidified atmosphere containing 5% CO2 at 37 °C. Collagenase I was from Sigma-Aldrich. Sulindac was obtained from Xiya Chemical CO., LTD (Chengdu, China). CD11b antibody conjugated with PerCP-Cy5.5, Gr-1 antibody conjugated with PE, CD3 antibody conjugated with PE, and CD8a antibody conjugated with FITC were from BD Pharmingen (San Diego, CA, USA). Antibody against F4/80 conjugated with PE and CD206 antibody conjugated with FITC, purified anti-mouse CD4 antibody and anti-mouse CD8a antibody were purchased from BioLegend (San Diego, CA, USA). Control isotype antibodies were from ZSGB-BIO company (Beijing, China).

### Tumor challenge and survival study

BALB/c mice and athymic nude female mice on a BALB/c background (8–10 week-old) were purchased from Beijing HFK Bioscience company (Beijing, China). All animals were maintained under specific pathogen-free conditions with individual ventilation. All animal experiments were approved and conducted in accordance with the Animal Care and Use Committee of Sichuan University. BALB/c mice were injected s.c. with 1 × 10^6^ 4T1 tumor cells in the right flank on day 0. Eight days after inoculation, tumor-bearing mice were treated intraperitoneally with 60 mg/kg sulindac for consecutive fourteen days. Tumour growth was monitored by measuring large (a) and short (b) length with a caliper every three days. Tumor volume was calculated as a × b^2^ × 0.52. For survival studies, BALB/c mice were injected s.c. with 1 × 10^6^ 4T1 tumor cells in the right flank. All mice were monitored until all death (more than over a 50 day period).

### *In vivo* T cell depletion

Depletion of T-cell subsets was achieved by injecting antimouse CD4 mAb or anti-mouse CD8 mAb. CD4+ or CD8+ T lymphocytes were selectively depleted in BALB/c mice by intraperitoneal administration of depleting anti-CD4 mAb (clone GK1.5, 300 μg per injection, n = 3) and anti-CD8 mAb (clone 2.43, 300 μg per injection, n = 3), respectively. An identical concentration and amount of isotype antibody was administered to IgG group. Antibodies were injected into tumor-bearing mice every five days with a total of three injections. The first injection was performed 2 days before sulindac therapy.

### Flow cytometry

Seven days after sulindac therapy, periphery blood, spleens and tumors were harvested. Red blood cells in periphery blood and spleens were lysed. Tumor single cells suspension were mechanically disrupted and then enzymatically digested with 1 mg/mL collagenase I. Cells in periphery blood and spleen as well as tumor cells were stained with CD11b-PerCP-Cy5.5, Gr-1-PE, F4/80-PE, CD206-FITC, CD3-PE, and CD8a-FITC for 30 min at 4 °C. All samples were aquired with FACSCalibur flow cytometer. Data analysis was performed using CellQuest software (BD Biosciences, San Jose, CA, USA).

### Immunofluorescence stain

Seven days after sulindac therapy, tumors were frozen-sectioned. Tumor tissues were stained with CD31-FITC overnight at 4 °C. Tumor vasculature were imaged and calculated under fluorescence microscope (Olympus).

### Real-time PCR

Total RNA was prepared from tumor tissues using a RNA isolation kit (Axygen) and reverse transcribed (TaKaRa) following the manufacturer’s protocols. cDNA was used to amplify S100A8, S100A9, S100A10, CCL-2, CSF-1, CSF-2, CXCL12, TNF-α, iNOS, IL-1β, IL-6, IL-12, VEGF, PlGF, arginase-1, TGF-β, versican, CCL3, CCL4, CCL5, CCL19 and CCL21. 18S RNA was used as reference control. PCR assay was performed on a CFX 96 real-time PCR thermocycler (Bio-Rad) using a kit (SYBR Premix EX Taq; TaKaRa). Primers used in PCR were shown in Supplemental Table S1.

### Statistical analysis

Data were expressed as the means ± SD. Statistics were analyzed by Student t test between two groups, and one-way ANOVA among more than three groups. Mouse survival was analyzed by Kaplan-Meyer analysis with a log-rank test. P values less than 0.05 was designated as statistical significant.

## Additional Information

**How to cite this article**: Yin, T. *et al*. Sulindac, a non-steroidal anti-inflammatory drug, mediates breast cancer inhibition as an immune modulator. *Sci. Rep.* **6**, 19534; doi: 10.1038/srep19534 (2016).

## Supplementary Material

Supplementary Information
