# Supplementary material for: Sulindac, a non-steroidal anti-inflammatory drug, mediates breast cancer inhibition as an immune modulator
Source: Sci Rep. 2016 Jan 18;6:19534. doi: 10.1038/srep19534 (PMC4725989; doi:10.1038/srep19534)
Supplement: Supplementary Information [file srep19534-s1.doc]

**Sulindac, a non-steroidal anti-inflammatory drug, mediates breast cancer inhibition as an immune modulator**

Tao Yin, Guoping Wang, Tinghong Ye, Yongsheng Wang

**Supplementary Figure S1.**

**
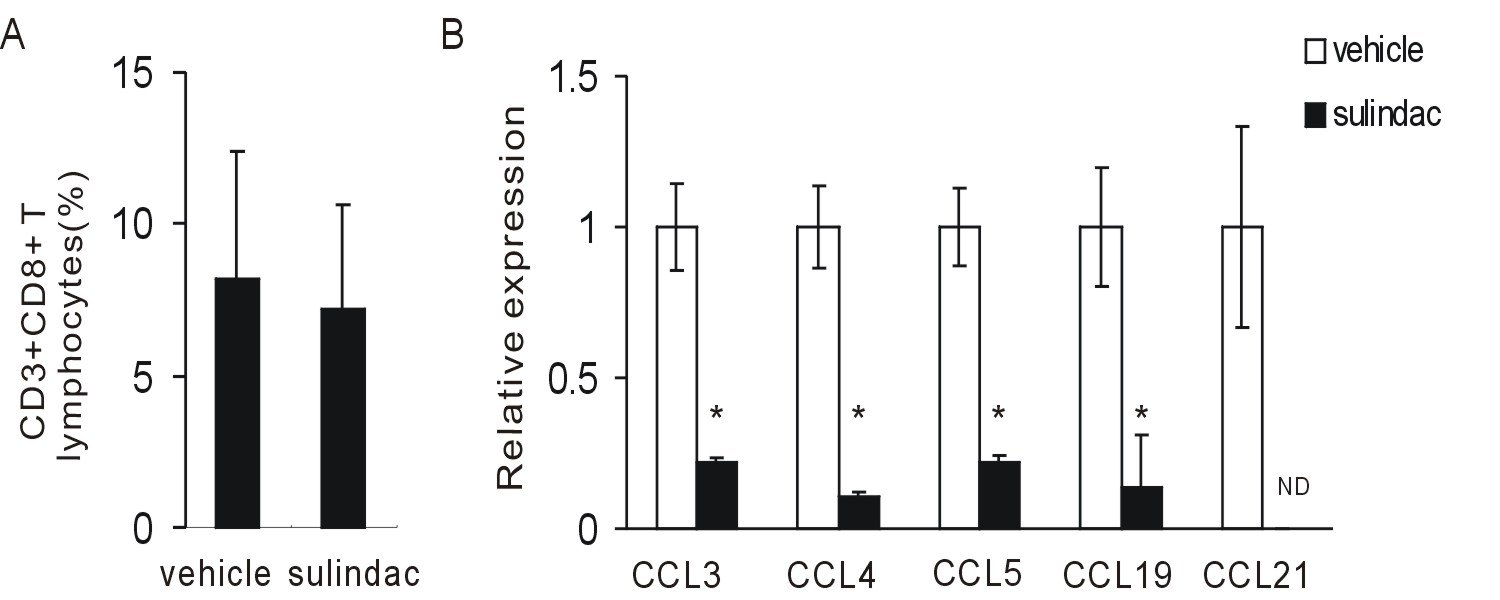
**

**Supplementary Figure S1. Sulindac did not increase the recruitment of CD8-positive T lymphocytes into tumor microenvironment.** A. Seven days after sulindac treatment, CD3+CD8+ T cells were evaluated in tumor tissues by flow cytometry. There was no increase of tumor-infiltrating CD8+ T cells after sulindac therapy. B. The levels of T cell recruiting chemokines, CCL3, CCL4, CCL5, CCL19 and CCL21, were analyzed by RT-PCR. ND, not detected. *P<0.05 vs control group.

**Supplementary Table 1. Primers sequence for RT-PCR.**

| gene | Forward | Reverse |
| --- | --- | --- |
| PIGF | CCCTGTCTGCTGGGAACAA | GCTGCGACCCCACACTTC |
| VEGF | CCACGTCAGAGAGCAACATCA | TCATCTCTCCTATGTGCTGGCTTT |
| iNOS | GGCAGCCTGTGAGACCTTTG | CATTGGAAGTGAAGCGTTTCG |
| Arginase-1 | GCTGTCTTCCCAAGAGTTGGG | ATGGAAGAGACCTTCAGCTAC |
| S100A8 | TGCGATGGTGATAAAAGTGG | GGCCAGAAGCTCTGCTACTC |
| S100A9 | CACCCTGAGCAAGAAGGAAT | TGTCATTTATGAGGGCTTCATTT |
| S100A10 | TGGAAACCATGATGCTTACGTT | GAAGCCCACTTTGCCATCTC |
| TNF-α | TCTTCTCATTCCTGCTTGTGG | GGTCTGGGCCATAGAACTGA |
| IL-1β | TGTAATGAAAGACGGCACACC | TCTTCTTTGGGTATTGCTTGG |
| IL-6 | GATGGATGCTACCAAACTGGAT | CCAGGTAGCTATGGTACTCCAGA |
| versican | TGGGGTGAGAACCCTGTATCGTTT | CCCATTGATATACTGCACTGACGG |
| TGF-β | CTCCCGTGGCTTCTAGTGC | GCCTTAGTTTGGACAGGATCTG |
| 18S rRNA | CGCCGCTAGAGGTGAAATTCT | CGAACCTCCGACTTTCGTTCT |
| IL-12 | CCTCAGTTTGGCCAGGGTC | CAGGTTTCGGGACTGGCTAAG |
| CXCL12 | GCGCTCTGCATCAGTGACGGTA | CCGTGCAACAATCTGAAGGG |
| CCL2 | CAGGTCCCTGTCATGCTTCT | GTCAGCACAGACCTCTCTCT |
| CSF-1 | GCCAGGCTTGTCTGTGGTGA | TAGCCAGGGAGGGCAGGAA |
| CSF-2 | CAACTCCGGAAACGGACTGTG | GCTGTGCCACATCTCTTGGTC |
| CCL3 | TGAAACCAGCAGCCTTTGCTC | AGGCATTCAGTTCCAGGTCAGTG |
| CCL4 | CCATGAAGCTCTGCGTGTCTG | GGCTTGGAGCAAAGACTGCTG |
| CCL5 | AGATCTCTGCAGCTGCCCTCA | GGAGCACTTGCTGCTGGTGTAG |
| CCL19 | CTGCCTCAGATTATCTGCCAT | TCATTAGCACCCCCCAGAGT |
| CCL21 | CCCTGGACCCAAGGCAGT | GGCTTAGAGTGCTTCCGGG |
